# Oxytocin Increases Heart Rate Variability in Humans at Rest: Implications for Social Approach-Related Motivation and Capacity for Social Engagement

**DOI:** 10.1371/journal.pone.0044014

**Published:** 2012-08-28

**Authors:** Andrew H. Kemp, Daniel S. Quintana, Rebecca-Lee Kuhnert, Kristi Griffiths, Ian B. Hickie, Adam J. Guastella

**Affiliations:** 1 School of Psychology, University of Sydney, Sydney, New South Wales, Australia; 2 Brain and Mind Research Institute, University of Sydney, Sydney, New South Wales, Australia; 3 CADE Clinic, Discipline of Psychiatry, Sydney Medical School, University of Sydney, Sydney, Sydney, New South Wales, Australia; Chiba University Center for Forensic Mental Health, Japan

## Abstract

**Context:**

Oxytocin (OT) plays a key regulatory role in human social behaviour. While prior studies have examined the effects of OT on observable social behaviours, studies have seldom examined the effects of OT on psychophysiological markers such as heart rate variability (HRV), which provides an index of individual’s *motivation* for social behaviour. Furthermore, no studies have examined the impact of OT on HRV under resting conditions, which provides an index of maximal *capacity* for social engagement.

**Objective:**

To examine the effects of OT on HRV measures in healthy male participants while at rest. OT was hypothesised to increase HRV, compared to placebo, and that the effects would be greatest for a non-linear measure of HRV (the detrended fluctuation scaling exponent).

**Methods:**

Twenty-one male participants were recruited for this study. Participants were non-smokers, not on any medications and reported no history of psychiatric illness, neurological disorder, or any other serious medical condition (e.g. diabetes, cardiovascular disease). The study employed a randomised, placebo-controlled, within-subject, crossover, experimental design.

**Main Outcome Measures:**

HRV was calculated from electrocardiography under a standardized, 10-minute, resting state condition.

**Results:**

As hypothesised, OT increased HRV and these effects were largest using the detrended fluctuation scaling exponent, a non-linear measure. These changes were observed in the absence of any change in state mood, as measured by the profile of mood states. Importantly, participants were unable to correctly guess which treatment they had been assigned at either of the two assessments.

**Conclusions:**

Together with the broader literature on OT and HRV, findings suggest that acute administration of OT may facilitate a fundamental psychophysiological feature of social behaviour, increasing *capacity* for social engagement. Findings also suggest that HRV changes may provide a novel biomarker of response to OT nasal spray that can be incorporated into research on response to treatment.

## Introduction

The mammalian neuropeptide, oxytocin (OT), plays a central regulatory role in human social behaviour and social cognition. OT nasal spray increases attention, encoding, and retrieval of social cues from memory, and it alters how social cues are appraised [1]. We have proposed a novel hypothesis for understanding the role of OT in human affect [2], [3] highlighting a key role for OT in the regulation of approach- and withdrawal-related, social behaviours. While social behaviour is associated with the *motivation* to approach or withdraw, this motivation may not always lead to overt, *observable behaviour*. One candidate psychophysiological marker of approach-related *motivation* is heart rate variability (HRV). Studies remain to be conducted that examine the effects of OT in humans on heart rate variability (HRV) at rest; a biomarker of an individual’s maximal capacity for social engagement. This is the objective of the present study.

OT may upregulate parasympathetic and/or reduce sympathoadrenal responses through a number of mechanisms. Receptors for OT are located in pathways regulating the myelinated vagus [4], which provides the neurophysiological substrates for social engagement. The myelinated vagus is regulated by the nucleus ambiguous, a brainstem nucleus that is also involved in regulation of the striated muscles of the face and head [4]. Receptors for OT are also located in pathways regulating the unmyelinated vagus [4]; OT receptors here may help to protect the autonomic nervous system during times of extreme stress avoiding for example, freezing behaviours and vasovagal syncope [4], [5]. OT may also impact on autonomic control through its influence on neural structures such as the amygdala; a region that expresses OT receptors in high density [6] and orchestrates complex autonomic patterns [7]. Neuroimaging research has demonstrated that the effects of OT are dependent on the nature of the stimulus, such that OT attenuates amygdala activation for fearful faces, but enhances activity for happy faces [8]. OT and its receptors are also present in cardiac tissue [9], indicating that peripheral mechanisms may also lead to changes in HRV. These mechanisms provide the neurophysiological substrates that underpin our social-approach/withdrawal hypothesis [2], [3], such that withdrawal-related behaviours are inhibited (anxiolysis), and approach-related social behaviours (social engagement) are increased.

HRV is the variability in consecutive heartbeats, such that increases in this measure reflect increased parasympathetic (inhibitory) control over sympathetic nervous system activity. HRV has traditionally been employed in studies examining cardiovascular risk: reduced HRV is associated with an increased risk of cardiovascular disease and sudden cardiac death [10]. Other studies have reported that increases in HRV are associated with positive emotions such as cheerfulness and calmness [11], and trait positive emotionality [12]; emotional states consistent with polyvagal theory [4], [5], which highlights a specific role for HRV in social engagement. By contrast, we have published a number of studies [13], [14] highlighting that large reductions in HRV are displayed in unmedicated, otherwise healthy patients with depression and anxiety; disorders characterised by reductions in approach and increases in withdrawal-related behaviours. However, null findings have also been reported and these may be due, in part, to reliance on time-domain or frequency HRV measures. It is now understood that the heart is not a periodic oscillator and conventional measures of HRV such as those based on time- and frequency-domain analysis may not be sufficiently sensitive to detect important (nonlinear) changes in heart rate time series [15], [16].

Heart rate in healthy participants are fractal because they display self-similar or scale-invariant fluctuations over a wide range of time series [17]. Fractal measures of HRV such as the detrended fluctuation scaling exponent do not measure the magnitude of the variability. Instead, the qualitative aspects and correlational features of heart rate behaviour are determined [17]. Consistent with this proposal we have recently demonstrated that non-linear measures of HRV [13], [14] and the detrended fluctuation analysis (DFA) technique [14] in particular, may be more sensitive to group differences. The current study will capitalise on the increased sensitivity of nonlinear measures by complementing HF HRV, an indicator of parasympathetic inhibition, with the detrended fluctuation scaling exponent (DFAα1), to determine whether OT increases the flexibility and adaptability of heart rate as measured by detrended fluctuation analysis.

Studies have seldom examined the capacity for OT to alter HRV and those that have report contradictory findings. Research on the socially monagomous prairie vole [18] reported that OT prevents the negative behavioural and autonomic consequences of long-term social isolation. Behaviours relevant to depression (i.e. reduced sucrose intake and increased immobility in the forced swim test), and autonomic parameters (i.e. increased basal heart rate and reduced HRV) that result from social isolation were prevented by 14 days of OT administration. A more recent study by the same authors [19] reported that 14-days of OT also reduces autonomic reactivity to acute stress. Both of these studies reported that autonomic effects of OT do not extend to prairie voles that had been paired with a female sibling. However, a study on healthy, college-age male and female human participants [20] demonstrated that OT increases high frequency (HF) HRV while participants were engaged in a variety of (non-stressful) activities. This study also reported decreased pre-ejection period, a marker of enhanced sympathetic control. Furthermore, no effects were observed on heart rate, blood pressure, pro-inflammatory cytokines, catecholamines or the HPA axis hormones highlighting the sensitivity of HRV measures to OT administration.

It remains unclear whether OT is able to increase HRV in a resting state in the absence of any external demands on participants. This is important because attentional demand decreases HRV [21], and OT may either attenuate or reverse these effects. Examining the effects of OT on HRV during rest will determine whether OT increases an individual’s *capacity* for social engagement. Increases in HRV at rest would have important implications for understanding the effects of OT on preparedness for social interaction and the ability to adapt to future environmental demands. The aim of the present study, therefore, was to examine the effects of OT on HRV measures in healthy male participants while at rest. We hypothesized that OT would significantly increase HRV, compared to placebo, and that the effects would be greatest for the detrended fluctuation scaling exponent, a non-linear measure of HRV. The sinus rhythm is controlled by multiple and complex neural mechanisms and non-linear analysis provides the means to capture this complexity embedded in heart rate time series. We expected changes to be observed in HRV in the absence of any change in state mood, as measured by the profile of mood states.

## Methods

### Participants

Twenty-one, non-smoking healthy male volunteers were recruited for this study and received either university course credit or reimbursement for their participation. Exclusion criteria were self-reported history of psychiatric illness, neurological disorder, or any other serious medical condition (e.g. diabetes, cardiovascular disease). Smokers were excluded from this study, as were those on psychotropic medications, including St. John’s Wort, which is often used as an antidepressant. Only males participated in this study to avoid the potential confounding effects of gender-dependent OT effects [22]. Participants were asked to abstain from caffeine, alcohol and illicit substances on the day of testing, and all food and drink (except water) for two hours prior to receiving the intranasal spray, as per previous studies [23]. The University of Sydney’s Human Research Ethics Committee provided ethical approval for this study and all participants gave written informed consent in accordance with National Health and Medical Research Council guidelines.

### Procedure

**Figure 1:**
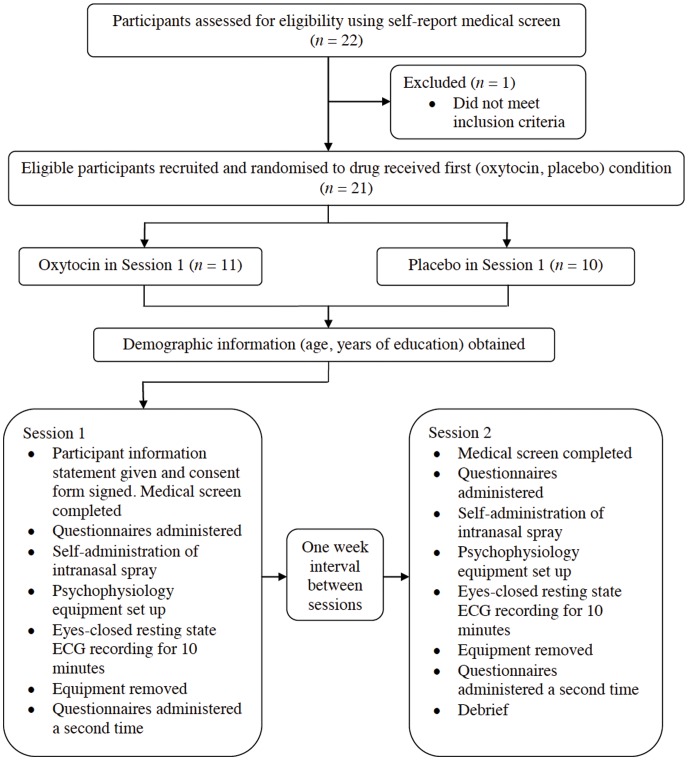
Participant flowchart.

This study employed a randomised, placebo-controlled, within-subject, crossover experimental design in order to investigate the effects of OT on HRV (see Fig. 1). Participants underwent two experimental sessions involving administration of either an OT or placebo intranasal spray at the first session, and the alternate spray at the second session. A one-week interval between sessions was implemented to minimise any carry-over effects of OT. The order of drug administration (OT first, placebo first) was counterbalanced across participants and participants were tested at the same time of day on both of their testing sessions (i.e., either in the morning: 0900–1200 h, or in the afternoon: 1400–1700 h), in order to minimize any circadian effects [24]. Prior to treatment administration, participants completed the Profile of Mood States (POMS) questionnaire [25], [26], a commonly used self-report measure of anxiety, anger, depression, confusion, fatigue and vigor, as per prior research on OT [27]. Participants rate each item on a 5-point Likert scale (0: “not at all” characteristic of how I feel right now; 4: “extremely” characteristic of how I feel right now). Participants were then administered a standard dose of 24 intranasal units (IU) (three puffs per nostril, each puff containing 4 IU) of either OT or placebo, which contained all ingredients except active OT.

Following administration of treatment, participants were seated in a dimly lit room and setup for the electrocardiogram (ECG) recording. Ag/AgCl electrodes were attached to each participant in a modified Lead-II formation: active electrodes were positioned on the right clavicle and left lower abdomen, and a reference electrode positioned at the tip of the nose. ECG recordings began 45 minutes after drug administration to coincide with the peak pharmacokinetics of OT. The ECG was recorded for ten minutes in a sitting, resting eyes-closed state using Contact Precision Instruments (Cambridge, Massachusetts) amplifiers, sampled at 500 Hz. Upon completion of recording, all equipment was removed and participants completed the POMS questionnaire for a second time.

### Data Analysis

ECG data from 4 participants could not be analysed due to difficulties with data collection, leaving a total of 17 participants for further analysis. All ECG data was manually inspected prior to analysis: data quality was high, consistent with resting-state recording conditions. R-R intervals were extracted from the ECG using LabChart software (version 5.1.1, ADInstruments, Australia), which were then imported into Kubios software (available at: http://kubios.uku.fi) for calculation of HRV measures including the high frequency (HF) component, expressed in normalized units, and the detrended fluctuation scaling exponent (DFAα1). Frequency domain analysis yields two or more peaks and the high frequency peak (0.15–0.4 Hz) was extracted for analysis as it the most commonly reported index of HRV, reflecting parasympathetic (vagal) control of heart rate [28]. Detrended fluctuation analysis technique determines the short-term, self-similar properties of the R-R interval time series. White Gaussian noise (a totally random signal) is reflected in a value of 0.5; a Brownian noise signal (a signal in which higher frequencies display decreased power) is reflected in a value of 1.5 [15]. Specific hypotheses were tested using two planned pair-wise comparisons (on HF and DFAα1) and a critical statistical threshold was set at *p*<0.05 (one-tailed). To determine whether OT had any effects on state mood, POMS data was analysed using a 2 Time (pre, post treatment) by 2 Treatment (OT, placebo) repeated measures ANOVA. Effect size measures (Cohen’s *d*) relating to the differences between treatment groups are reported when significant.

## Results

### Participant Characteristics

Participants were aged between 19 and 42 years (M = 24.48, SD = 5.05), with years of education ranging from 13 to 24 years (M = 16.57, SD = 2.93). Consistent with prior studies [27], OT did not impact on anxiety, anger, depression, confusion, fatigue, or vigor (all p’s >0.48). Participants were unable to correctly guess which treatment they had been assigned at either of the two assessments. The percentage of participants guessing they received OT did not differ by drug condition for session 1 (30% receiving placebo versus 18% receiving OT, p = 0.64, Fisher’s exact test), nor for session 2 (64% receiving placebo versus 30% receiving OT, p = 0.19, Fisher’s exact test). In total, 5 participants on OT and 13 on placebo reported side effects. Side effects over the two sessions included slight headache (N = 8), drowsiness (N = 7), feeling relaxed (N = 2) and tingling in extremities (N = 1).

### Heart Rate Variability

**Figure 2:**
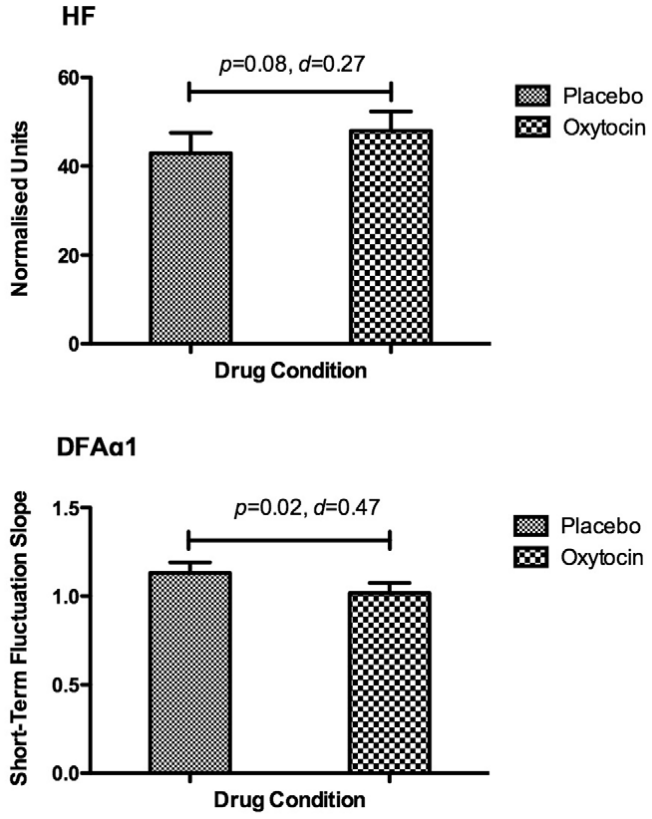
Heart rate variability (HRV) findings for high frequency (HF) – the most commonly reported measure of HRV – and the detrended fluctuation scaling exponent (DFAα) – a non-linear measure of HRV that is more sensitive to the impact of OT. Data presented are means ± standard errors of the mean (SEM).

As hypothesised, HRV was increased under OT relative to placebo conditions for both DFAα1 [*t*(16) = 2.13, *p* = 0.02, *d* = 0.47] and HF [*t*(16) = 1.468, *p* = 0.08, *d* = 0.27] (See Fig. 2). Although participants display a lower DFAα1 value than those given placebo, this finding indicates a more random signal consistent with an increase in HRV. Kolmogorov-Smirnov and Shapiro-Wilk tests of normality both confirmed that the distributions of DFAα1 and HF were normal.

## Discussion

Findings supported the hypothesis that OT acts to increase HRV in the absence of any external demands on participants, consistent with the concept that OT (directly or indirectly) increases ones *capacity* for social approach-related behaviour. We also demonstrate that the impact of OT is greatest in non-linear measures of HRV consistent with the proposal that non-linear measures may be more sensitive to group differences [13] and have more clinical relevance than conventional measurements of HRV [16]. The present study has a number of methodological strengths including a randomised, placebo-controlled, within subject, crossover experimental design; examination of non-linear measures of HRV; and inclusion of only male participants to avoid the potential confounding effects of gender.

A number of theories provide a theoretical framework in which the effects of OT on HRV may be understood. We have previously proposed [2], [3] that OT may serve to increase social approach-related motivation, and decrease withdrawal-related motivation. This theory has a number of features; the most relevant for the current study is that motivation involves energizing and directing action, which may or may not manifest itself as an observable behaviour. In the current study we observed OT to increase HRV, while no changes were observed on self-report measures of mood states. These findings are interpreted in terms of increasing the motivation and capacity for approach-related behaviour. The polyvagal theory [4], [5] provides the theoretical framework for understanding how increases in HRV may be related to the motivation for approach-related behaviour. The polyvagal theory focuses on the phylogenetic shift from reptiles to mammals involving specific changes to the vagal pathways regulating the heart [29]. Only mammals have a myelinated vagus – originating in the nucleus ambiguous – and through the process of evolution, the brainstem nuclei became integrated with the muscles of the face and head. These connections provide the neurophysiological substrates for social engagement for which approach-related motivation is essential. Our findings therefore have important implications for understanding the physiological correlates underpinning approach-related motivation.

While OT may serve to increase ones capacity for approach-related motivation, this capacity need not be a conscious process. Indeed, our study observed no change in anxiety, anger, depression, confusion, fatigue, or vigor as assessed using the POMS [25], [26]. The polyvagal theory [29] proposes an automatic, subcortical mechanism – neuroception – that is capable of identifying environmental and visceral features that are safe, dangerous or life-threatening. Neuroception is triggered by feature detectors in the temporal cortex, which respond to voice prosody, facial expressions and hand movements. OT may serve to increase ones sensitivity to such cues. In this regard, we have observed OT to increase ones gaze to the eye region of human faces [23], which may serve to enhance emotion recognition, interpersonal communication and social approach. Neuroception may also be triggered by afferent feedback from the viscera; this visceral feedback represents a major mediator of the extent to which social engagement will take place. Reduced HRV observed in, for example, the mood and anxiety disorders [13], [30] will compromise the capacity to detect positive social cues and engage with others. By contrast, we show here that OT increases HRV, which will lead to increased flexibility and adaptiveness to the environment.

Although there has been a proliferation of OT research, relatively few studies have focused on autonomic responses. This is an important area of study considering the health benefits associated with increased HRV. Investigation of the impact of OT on HRV also provides an opportunity to understand effects on *motivation* for social behaviour. An earlier study reported that OT increases autonomic (parasympathetic and sympathetic) cardiac control [20] while participants were engaged in a variety of (non-stressful) activities. Here we observed increases in parasympathetic control (increased HF HRV, at trend levels), and a significant reduction in the nonlinear measure DFAα1 while participants in the absence of any external demands on participants. Tulppo and colleagues [17] have characterised reductions in DFAα1– a finding we report here – as coactivation of sympathetic and vagal outflow; consistent with that reported by Norman and colleagues [20]. Our findings therefore provide support for the findings reported by Norman and colleagues but extend their findings to the impact of OT on HRV during a quiet resting state.

As well as providing support for our social-approach/withdrawal hypothesis, our findings shed further light on a more comprehensive model of motivation. A recent review of animal and human neuroendocrinology [31] highlights that approach and withdrawal motivation provide an important framework for understanding affect, cognition and behaviour across a variety of domains. However, an important distinction is made between approach-motivated appetitive states that increase capacity for action versus a quiescent state associated with positive feelings of warmth and calm in which motivation to act is absent. The authors highlight that approach motivation and quiescence should be distinguishable in terms of autonomic responses. They suggest that approach motivation would be associated with increased sympathetic activity, while quiescence would be associated with parasympathetic activity. Norman and colleagues [20] reported that OT increases both parasympathetic and sympathetic control. Consistent with this, we show here that OT decreases the detrended fluctuation scaling exponent, a finding that is associated with coactivation of sympathetic and vagal outflow [17]. We suggest therefore that OT facilitates approach-related behaviour rather than quiescence.

Our findings suggest that heart rate variability changes may provide a novel biomarker of OT nasal spray influence in humans that can be incorporated into circuitry models. We have argued for the need to adopt a circuitry approach to understand the impact of OT nasal spray in humans [1]. HRV may have value in predicting therapeutic response to intervention, or even the direction of the response to intervention. This possibility needs to be examined in future research. Future studies should consider collecting HRV data before and after drug administration to minimise within-subject variability of HRV and maximise observable effects. On the basis of our research here, and that of others [20], it would seem prudent to incorporate heart-rate variability measures into clinical studies to determine utility as a potential marker of response to intervention.

The recent proliferation of human research on OT highlights a critical role for OT in the regulation and facilitation of approach-related motivation, which led to the proposal of our social-approach/withdrawal hypothesis. A limitation of our study is that no psychological assessment was conducted to distinguish between the different hypotheses that have been proposed for the impact of OT [3]. Future studies should consider using tasks that involve emotional experience and that directly compare the specific hypotheses. Regardless, the present study identifies HRV as a potential marker of response to OT, and suggests that OT may increase approach-related *motivation* and the *capacity* for social engagement. As the potential therapeutic implications of OT are increasingly explored, this study provides important evidence for how OT may exert its salubrious effects, particularly in those disorders characterized by impairments in social cognition and social engagement such as autism, affective disorders and substance dependence. In conclusion, the findings of the present study indicate that intranasal OT administration increases HRV at rest and that these effects may be greatest for non-linear measures of HRV.
